# Efficacy of Angiotensin Receptor-Neprilysin Inhibitor and Its Renal Outcome in Heart Failure Patients: A Systematic Review of Randomized Clinical Trials

**DOI:** 10.7759/cureus.54501

**Published:** 2024-02-19

**Authors:** Naiela E Almansouri, Saloni Bakkannavar, Youmna Faheem, Amisha Jaiswal, Kainaat Shergill, Kusalik Boppana, Tuheen Sankar Nath

**Affiliations:** 1 Internal Medicine, California Institute of Behavioral Neurosciences and Psychology, Fairfield, USA; 2 Internal Medicine, University of Tripoli, Tripoli, LBY; 3 Pediatrics, California Institute of Behavioral Neurosciences and Psychology, Fairfield, USA; 4 Surgery, Maharishi Markandeshwar Institute of Medical Sciences and Research, Mullana, IND; 5 Medicine, California Institute of Behavioral Neurosciences and Psychology, Fairfield, USA; 6 Surgical Oncology, Tata Medical Centre, Kolkata, IND

**Keywords:** lcz696, neprilysin inhibition, heart failure, renal function, sacubitril/valsartan

## Abstract

Heart failure (HF) is a major cause of morbidity and mortality and imposes a significant financial burden on healthcare systems globally. Angiotensin receptor-neprilysin inhibitor (ARNI), a novel neuroendocrine inhibitor, is frequently used in treating HF. However, there is still limited understanding regarding how it compares to other neuroendocrine inhibitors, such as angiotensin-converting enzyme inhibitors (ACEis) and angiotensin receptor blockers (ARBs). The purpose of this research is to present the most recent data regarding the efficacy and renal impact of ARNIs in the treatment of HF in comparison to ACE inhibitors and ARBs. Several large-scale randomized controlled trials (RCTs) have recently been conducted to evaluate the benefits of this drug in patients with different types of HF, regardless of their renal status. We searched multiple databases, including PubMed, PubMed Central (PMC), and Google Scholar, to find relevant RCTs. The efficacy outcome was a composite of the rate of death from cardiovascular causes, the frequency of HF hospitalizations (HFH), and alterations in N-terminal pro-brain natriuretic peptide (NT-proBNP) levels. The renal outcome was impairment of renal function. This systematic review analyzed large-scale RCTs involving 17,327 participants, with an average follow-up time of approximately 2.9 years. sacubitril/valsartan showed notable improvements compared to ACEis and ARBs in the following areas: reduction in NT-proBNP levels, prevention of further deterioration in renal function, and decreased hospitalizations for HF. Interestingly, there is no increased risk of mortality from cardiovascular causes with sacubitril or valsartan.

## Introduction and background

Heart failure (HF) was identified as an increasing epidemic nearly thirty years ago [1]. The current global burden of HF is approximately 64 million [1]. The prevalence of HF exceeds 1% in numerous countries and regions worldwide [2]. HF has experienced significant growth over the past decade in both developed and developing countries [2]. Notably, the prevalence rate rises with age, reaching more than 10% among adults over 70 [3]. In addition, it has the potential to place a significant economic burden on all nations globally [4].

HF is a clinical syndrome that occurs when the heart's ability to pump blood or fill with blood is impaired because of various cardiac diseases affecting its function or structure [4]. It represents a terminal stage of different types of cardiovascular diseases (CVDs), earning it what is known as the "last battlefield" of CVDs [5,6]. HF comprises patients who are classified based on their symptoms and ejection fraction (EF). This includes patients with HF with reduced EF (left ventricular EF ≤ 40%; HFrEF), mildly reduced EF (EF between 40% and 49%; HFmrEF), and preserved EF (EF ≥ 50%; HFpEF) [7]. According to studies primarily on hospitalized patients, approximately 50% of those with HF are believed to have HFrEF, and 50% have HFpEF, HFmrEF [5].

Numerous pharmaceutical interventions, including angiotensin-converting enzyme (ACE) inhibitors, calcium channel blockers (CCBs), beta-blockers, and angiotensin receptor blockers (ARBs), have been suggested for the management of HF; however, none of them has demonstrated significant efficacy [8-10]. The European Society of Cardiology updated its guidelines for managing HF in 2016 [7], incorporating LCZ696, a novel drug class, into the therapeutic algorithm. Sacubitril, an inhibitor of neprilysin, is combined with valsartan, an angiotensin receptor antagonist. Neprilysin inhibition elevates vasoactive peptide concentrations, reducing sodium retention, detrimental remodeling, and vasoconstriction. Valsartan was selected for combination therapy to inhibit the renin-angiotensin system (RAS) and, when compared to ACE inhibitors, to reduce the risk of angioedema [11].

The current novel medication is recommended as an alternative to ACE inhibitors for patients diagnosed with HF with reduced EF (HFrEF). This recommendation applies specifically to cases where patients, despite receiving optimal treatment, including a beta-blocker, ACE inhibitor, and mineralocorticoid receptor antagonist, still experience symptoms (NYHA classification II-III) [12].

The European Cardiac Society continues to endorse the use of angiotensin receptor-neprilysin inhibitors (ARNIs) as a viable alternative to angiotensin-converting enzyme inhibitors (ACEis) in appropriate patient populations as of 2023 [13]. The pharmaceutical compound in question is currently in its early stages of development and is undergoing rigorous testing across several demographic groups [12]. Our research centers around studying patients diagnosed with different types of HF: HFrEF and HFpEF.

Incorporating ARNI may emerge as a noteworthy component of clinical guidelines in the future, hence offering substantial advantages to patients. This systematic review aimed to assess the effectiveness of ARNI therapy and its impact on renal outcomes in various types of HF based on previous randomized clinical trials.

## Review

Methods

The systematic review was done and reported in accordance with the principles outlined in the Preferred Reporting Items for Systematic Reviews and Meta-Analyses (PRISMA) 2020 [14]. We strictly adhere to the guidelines outlined by this approach. The study question aimed to determine the effectiveness of ARNI and its renal impact on patients with HF.

Search Sources and Search Strategy

We conducted an in-depth search and retrieval of relevant publications using four significant electronic databases in the domain of research literature. We gathered our data using PubMed, PubMed Central (PMC), Cochrane Library, and Google Scholar between September 10 and 15, 2022. We created appropriate keywords using Boolean operators and the Medical Subject Headings (MeSH) strategy to find the necessary articles. These selected keywords enabled us to identify the relevant and significant papers that showcase the evidence supporting ARNI's effectiveness and outcome in HF. We searched for articles in PubMed Central and Cochrane Library using these keywords, individually and in combination. The keywords are “Sacubitril/Valsartan, Renal function, Heart failure, Neprilysin inhibition, LCZ696”. The details of the search strategy are listed in Table 1.

**Table 1:** The strategy of the database search with their respective filters and results

Database	Search Strategy	Filter Applied	Results
PubMed Central and Medline	"Treatment Outcome"[Majr] OR Efficacy OR Effectiveness AND "sacubitril and valsartan sodium hydrate drug combination" [Supplementary Concept] OR “Angiotensin receptor-neprilysin inhibitor” OR ARNI OR LCZ696 OR “Sacubitril/Valsartan” AND ( "Renal Insufficiency/blood"[Majr] OR "Renal Insufficiency/classification"[Majr] OR "Renal Insufficiency/complications"[Majr] OR "Renal Insufficiency/diagnosis"[Majr] OR "Renal Insufficiency/drug therapy"[Majr] OR "Renal Insufficiency/epidemiology"[Majr] OR "Renal Insufficiency/etiology"[Majr] OR "Renal Insufficiency/mortality"[Majr] OR "Renal Insufficiency/pathology"[Majr] OR "Renal Insufficiency/physiopathology"[Majr] OR "Renal Insufficiency/therapy"[Majr] OR "Renal Insufficiency/urine"[Majr] ) OR Renal outcome OR Renal function OR Renal effect OR Renal side effect AND ( "Heart Failure/blood"[Majr] OR "Heart Failure/chemically induced"[Majr] OR "Heart Failure/classification"[Majr] OR "Heart Failure/complications"[Majr] OR "Heart Failure/diagnosis"[Majr] OR "Heart Failure/drug therapy"[Majr] OR "Heart Failure/mortality"[Majr] OR "Heart Failure/pathology"[Majr] OR "Heart Failure/physiopathology"[Majr] OR "Heart Failure/prevention and control"[Majr] OR "Heart Failure/urine"[Majr] ) OR Heart failure OR CHF OR “Congestive heart failure”	Free full text, Human, English, Adult population	261
Cochrane Library	Keywords: “myocardial failure” AND “Sacubitril-Valsartan”	2012-2023 Open access	8
Google Scholar	Keywords: “Angiotensin–Neprilysin Inhibition” AND “left ventricular ejection fraction” OR “LVEF”	2012-2023	17

Study Selection and Eligibility Criteria

Each article was meticulously reviewed to eliminate any duplicates. The selected articles were then thoroughly examined to identify and exclude irrelevant ones. This was done by carefully reviewing the abstract, title, and subject headings of each article. Following the selection of the papers, each one underwent a quality assessment using the PRISMA Checklist 2020 [14]. Additionally, the full text of each article was carefully read to ensure it met the selection criteria. Subsequently, we implemented the inclusion and exclusion criteria to further narrow down the choice of papers before commencing the study. The systematic review was done in adherence to ethical standards.

Inclusion Criteria

We included articles published in English literature over the past 12 years based on human studies. Next, we pinpointed papers focusing on the adult population in relation to HF and added papers related to ARNI. Our eligibility requirements based on the PICO (population, intervention, control, and outcomes) study criteria include patient, population, problem, intervention/exposure, comparison, and outcome.

Exclusion Criteria

**Figure 1:**
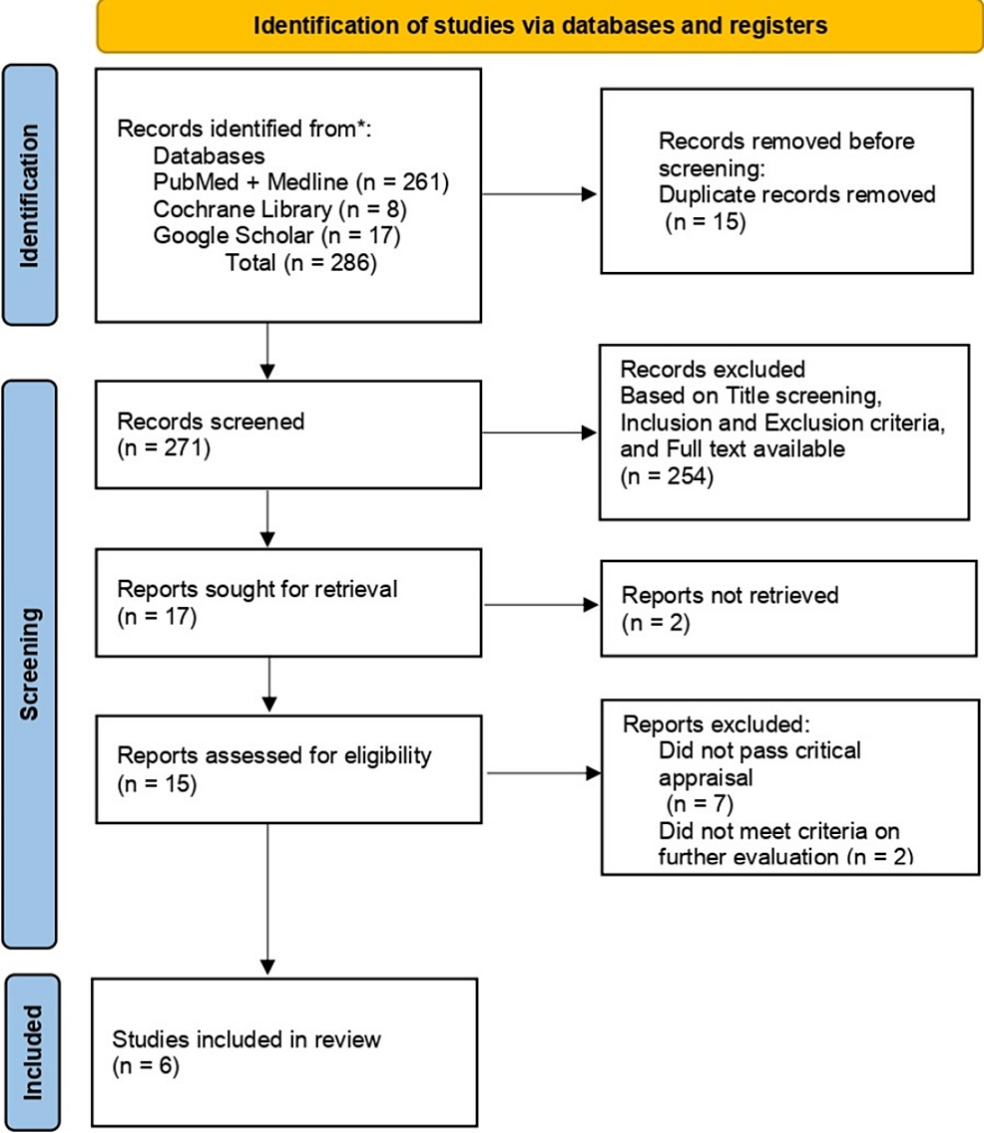
PRISMA diagram detailing the study identification and selection process PRISMA, Preferred Reporting Items for Systematic Reviews and Meta-analysis

In our paper, we decided to exclude certain types of sources. These include gray literature, letters to the editor, animal studies, unpublished literature, and papers discussing the pediatric population (Figure 1).

Risk of Bias and Quality Assessment

We independently assessed the quality of each publication using the Cochrane bias assessment method for randomized controlled trials (RCTs). This approach helps to minimize the potential for bias. We checked every article for seven types of bias: allocation concealment, random sequence generation, incomplete outcome data, selective reporting, blinding of participants and outcome assessment, and other biases. We ranked each type of bias as low, high, or unclear (Table 2).

**Table 2:** Cochrane appraisal +, low risk of bias; -, high risk of bias; ?, unclear risk of bias; N/A, not assessed

Cochrane appraisal	Random Sequence Generation	Allocation Concealment	Blinding of Participant	Blinding of Outcome Assessment	Incomplete Outcome Data	Selective Reporting	Other Bias
PARAMOUNT-HF, 2012 [15]	+	+	+	+	+	+	?
PARADIGM-HF, 2014 [16]	+	?	+	+	+	+	+
PIONEER-HF, 2019 [17]	+	?	+	+	+	+	+
PARAGON-HF, 2019 [18]	+	+	+	+	+	+	+
PARALLAX-HF, 2020 [19]	+	+	+	+	+	+	+
PARAGLIDE-HF, 2023 [20]	+	+	+	+	+	+	+

Results

After searching through different online databases and libraries, we selected 286 publications for our research evaluation. These publications were then reviewed for duplicates and assessed against the inclusion and exclusion criteria we devised (focusing on papers on ARNI and research studies on HF, while eliminating grey literature, pediatric population studies, and letters to the editor). Fifteen duplicate articles were excluded from the initial total of 286 articles. Additionally, 254 articles were filtered out due to strict title screening, irrelevance to the inclusion and exclusion criteria, lack of full-text availability, and their being off-topic. After applying a rigorous filtering process, 17 articles were included in the study. Of these, two articles were unavailable, seven failed the critical appraisal, and two did not meet the criteria for further evaluation.

Consequently, only six articles remained, all of which were double-blinded RCTs that satisfied our predefined criteria. The findings are also illustrated in Figure 1. The research involved both adult males and females who had been previously diagnosed with HF, regardless of their renal status. ARNI and various RAS inhibitors (enalapril, valsartan) were used at different dosages. Five of the six studies selected demonstrated positive results in reducing NT-proBNP levels, cardiovascular hospitalizations, and the incidence of composite renal impairment. However, the PARAGON-HF 2019 trial did not achieve its primary outcome of cardiovascular death and hospitalization for HF (HFH) across the entire study population. Nevertheless, a subgroup analysis found that individuals with a left ventricular EF below the study's median value of 57% experienced a significant reduction in HFH. These results were obtained during the follow-up period mentioned in Table 3.

**Table 3:** RCT study design HFpEF, heart failure preserved ejection fraction; HFrEF, heart failure reduced ejection fraction; HFmrEF, heart failure mildly reduced ejection fraction; WHF, worsening heart failure; CV death, cardiovascular death; NT-proBNP, N-terminal pro-B-type natriuretic peptide; Sac/val, sacubitril/valsartan; Scr, serum creatinine; eGFR, estimated glomerular filtration rate; UACR, urine albumin creatinine ratio; NA, not available

Study Included	PARAMOUNT-HF, 2012 [15]	PARADIGM-HF, 2014 [16]	PIONEER-HF, 2019 [17]	PARAGON-HF, 2019 [18]	PARALLAX-HF, 2020 [19]	PARAGLIDE-HF, 2023 [20]
Drug	Sac/val	Sac/val	Sac/val	Sac/val	Sac/val	Sac/val
Control	Valsartan	Enalapril	Enalapril	Valsartan	IMT	Valsartan
Population	HFpEF	HFrEF	HFrEF	HFpEF	HFpEF	HFmrEF or HFpEF following stabilization from a WHF event
Number of participants	149	8442	882	4822	2566	466
Follow up	8 months	27 months	2 months	35 months	5.5 months	20 months
Primary outcome	Change in NT-proBNP	CV death or heart failure hospitalization	Time-averaged proportional change in NT-proBNP	CV death or heart failure hospitalization	Change in NT-proBNP and change in the 6-minute walk distance	Change in NT-proBNP
Definition of decline in renal function	Scr ≥ 0.3 mg/dL ↑ and/or >25% ↑ between two time points	end-stage renal disease, 50% ↓eGFR, 30↓GFR to <60 mL/min per 1.73 m^2^	Scr ≥ 0.5 ↑mg/dL (≥44 µmol/L), 25%↓GFR	death from renal failure, end-stage renal disease, or 50%↓eGFR	Acute kidney injury, renal failure, renal impairment, or renal injury	end-stage renal disease or ≥50% decline in eGFR relative to baseline
Baseline Serum Cr (mg/dL)	NA	1.13 ± 0.3 vs 1.27 ± 0.03	1.28 (1.07-1.51) vs 1.27 (1.05-1.50	1.1 ± 0.3 VS 1.1 ± 0.3)	NA	1.3 (1.0-1.6) vs 1.2 (1.0-1.5)
Baseline eGFR (mL/min per 1.73 m^2^)	66.5 ± 19.4 vs 64.3 ± 21.3	NA	58.4 (47.5-71.5) vs 58.9 (47.4-70.9)	63 ± 19 vs 62 ± 19	62.5 (20.2) vs 62.7 (19.6)	47.4 (36.4-62.2) vs 51.1 (39.4-64.8)
Conclusion	Sac/val was found to have a greater impact on reducing NT-proBNP levels compared to valsartan. Sac/val showed a positive effect on eGFR preservation, although it led to an increase in UACR	Sac/val revealed higher efficacy to enalapril in reducing the risks of death of HFH. It exhibited a slower decrease rate in the eGFR compared with enalapril alone, despite causing a modest increase in UACR	Sac/val resulted in a more pronounced decrease in NT-proBNP levels compared to enalapril. The incidence of worsening renal function showed no significant difference between the two groups	There was no significant difference observed between the two groups in total HFH and death from CV causes, and they were less likely to have increases in creatinine	Sac/val demonstrated a greater reduction in NT-proBNP compared to valsartan after 12 weeks of treatment	Sac/val led to a greater reduction in NT-proBNP and potentially improved clinical outcomes compared to valsartan. It showed numerically fewer cardiovascular and renal events but a higher incidence of symptomatic hypotension

Discussion

This systematic review evaluated ARNI's benefits and renal outcomes in HF patients, regardless of their kidney function. After conducting an extensive online search, we carefully chose six studies. These studies were double-blinded, RCTs with a combined sample size of 17,327 participants.

Mechanism, Efficacy, and Renal Outcomes of ARNI

Angiotensin receptor neprilysin inhibitors (ARNi) are a novel pharmacological category characterized by the dual action of inhibiting neprilysin and blocking angiotensin II receptor type 1 (AT1). The intervention has demonstrated an enhancement in ventricular function by mitigating the adverse consequences of renin-angiotensin-aldosterone system (RAAS) activation and inhibiting neprilysin to degrade endogenous natriuretic peptides. Furthermore, it has exhibited favorable outcomes in individuals with mild-to-moderate arterial hypertension and HF [21]. Simultaneously blocking an ACE inhibitor or an angiotensin receptor blocking agent like valsartan, an NEP inhibitor, not only interrupts the RAS but also enhances the availability of bradykinin, nitric oxide, and prostacyclin. This unique dual action consistently magnifies the overall hypotensive effect of the blockade [22].

The efficacy outcome

Reducing Cardiovascular Death and Hospitalization for HF

The effect of sacubitril/valsartan on reducing cardiovascular death and the rate of hospitalization among patients with HF was reported in two randomized clinical trials, PARADIGM-HF and PARAGON-HF, representing 13,264 patients. The PARAGON-HF trial did not achieve the primary outcome of cardiovascular death and HFH in the entire study population [18]. However, a subgroup analysis found a significant decrease in HFH among individuals with a left ventricular EF below the study's median value of 57%. The hazard ratio for this subgroup was 0.78 (95% confidence interval (CI): 0.64 to 0.95) [17]. The PARADIGM-HF trial has shown a reduction in a hazard ratio of 0.80 (95% CI: 0.73 to 0.87; P<0.001) and 0.79 (95% CI: 0.71-0.89; P<0.001) were deaths due to cardiovascular causes, and HFH; respectively [16]. The 95% CI, P-value, and hazard ratio for each trial are shown in Table 4.

**Table 4:** RCT versus placebo RCT, randomized controlled trial; CI, confidence interval; CV death, cardiovascular death; HHF, hospitalization for heart failure; LVEF, left ventricular ejection fraction

RCT Versus Placebo	Hazard Ratio for Primary Outcome	95% CI, P-Value
PARADIGM-HF (2014) [16]	0.80 (CV death); 0.79 (HHF)	0.73-0.87, <0.001; 0.71-0.89, <0.001
PARAGON-HF (2019) [18]	0.78 (subgroups LVEF< 57%)	0.64-0.95,0.01

Reducing NT-ProBNP

This outcome was reported in four randomized clinical trials, PARAGLID-HF, PARALLAX-HF, PIONEER-HF, and PARAMOUNT-HF, representing 4063 patients. Among the patients receiving sacubitril-valsartan (LCZ696), the outcome showed a significant reduction of NT-proBNP in these four trials. Table 5 displays the 95% CI, P-value, and ratio of change for each trial.

**Table 5:** RCT versus placebo RCT, randomized controlled trial; CI, confidence interval

RCT Versus placebo	Ratio of Change for Primary Outcome	95% CI, P-value
PARAMOUNT-HF (2012) [15]	0.77	0.64-0.92, 0.005
PIONEER-HF (2019) [17]	0.71	0.63-0.81, 0.001
PARALLAX-HF (2020) [19]	0.48	0.80-0.88, 0.001
PARAGLIDE-HF (2023) [20]	0.5	0.73-0.999, 0.049

The renal outcome

There is mounting evidence regarding the cardiovascular advantages of sacubitril/valsartan in patients diagnosed with heart failure, characterized by reduced ventricular function and preserved EF [23]. Nonetheless, the impact of this medication on renal function exhibits inconsistency.

HFpEF Patients

The renal outcome was reported in a study on patients with HFpEF in four randomized clinical trials, encompassing 8005 individuals.

The PARAMOUNT-HF trial had 149 patients; patients were randomly given sacubitril/valsartan or valsartan, and the follow-up was eight months. Over 36 weeks, there was a greater decrease in the estimated glomerular filtration rate (eGFR) in the valsartan group (sac/val, -1.6 mL/min per 1.73 m² vs valsartan, -5.2 mL/min per 1.73 m²; p=0.007) and a more significant increase in the urinary albumin creatinine ratio (UACR) in the group sacubitril/valsartan (sac/val, 1.9 mg/mmol at baseline, 2.9 mg/mmol at week 36; valsartan, 2.0 mg/mmol at baseline, 2.0 mg/mmol at week 36; p=0.02) [15]. Voors et al. conducted a secondary analysis of the PARAMOUNT trial and found that sacubitril/valsartan exhibited eGFR preservation compared to valsartan therapy in patients with HFpEF over 36 weeks. However, it was also associated with increased UACR [24].

The PARAGON-HF included 4822 patients who randomly received sacubitril/valsartan or valsartan for 2.9 years. The baseline eGFR <60 mL/min/1.73 m^2^ showed a significant relative risk (RR) of 0.79 (95% CI: 0.66-0.95), while the RR for eGFR of 60 mL/min/1.73 m^2^ was not significant at 1.01 (0.80-1.27). These findings emphasize the importance of renal function in the outcome of HFpEF, which is better preserved by sacubitril-valsartan [18]. Similarly, the second analysis of the PARAGON-HF trial found that sacubitril/valsartan decreased the risk of renal events and delayed the decline in eGFR in patients with HFpEF compared to valsartan [25].

The PARALLAX-HF was conducted on around 2566 patients randomly assigned the sacubitril/valsartan versus valsartan or enalapril for 5.5 months. At week 24, it was observed that the group receiving sacubitril/valsartan had a significantly lower monthly change from baseline (slope) in eGFR compared to the group receiving individualized medical therapy (−0.25 vs −0.43 mL/min/1.73 m^2^ respectively, P = 0.02). The rate at which the estimated glomerular filtration rate (eGFR) declined annually was -2.95 mL/min/1.73 m^2^ in the group treated with sacubitril/valsartan and -5.14 mL/min/1.73 m^2^ in the comparator group. This reflects a statistically significant adjusted mean treatment difference of 2.19 mL/min/1.73 m^2^ (95% CI: 0.41 to 3.97; P = 0.01) [19].

The PARAGLID-HF was done with 466 patients; patients with mild reduced heart failure or preserved heart failure following stabilization from a worsening heart failure event were randomly given sacubitril/valsartan or valsartan for 1.6 years. Sac/Val had a lower incidence of renal function deterioration (odds ratio (OR): 0.61; 95% CI: 0.40-0.93) and a higher incidence of symptomatic hypotension (OR: 1.73; 95% CI: 1.09-2.76) compared to Val [20].

HFrEF Patients

The renal outcomes were documented in a study conducted on patients diagnosed with HFrEF in two randomized clinical trials comprising 9324 individuals.

The PARADIGM had the largest group of people (8442) in all conducted trials examined; patients were randomly assigned sacubitril/valsartan or enalapril for 2.9 years. The study results demonstrated that sac/val exhibited superiority over the enalapril group in terms of the decline in renal function, with a hazard ratio (HR) of 0.86 (95% CI: 0.65 to 1.13; P=0.28) [16]. In the secondary analysis of the PARADIGM-HF trial, the decline in eGFR induced by sacubitril/valsartan was slower compared to enalapril alone. However, it did cause a slight increase in UACR among individuals with HFrEF and a baseline eGFR of 70 mL/min/1.73 m^2^ [26].

The PIONEER included 882 patients who randomly received sacubitril/valsartan or enalapril for two months. The study found no significant differences between the sacubitril-valsartan group and the enalapril group regarding renal impairment, with an RR of 0.93 (95% CI: 0.67 to 1.28) [12].

Limitations of the study

The longest follow-up period among the trials included in this analysis was 35 months; therefore, it is not possible to determine the long-term effect of sacubitril/valsartan on renal outcomes due to the absence of long-term data. In addition, the included trials had a follow-up period of at least two months, which may not accurately reflect the final outcome. In this review, we only included articles written in English. However, this approach may have caused us to overlook valuable studies in other languages that could have enhanced the strength of our review. We also could not fully evaluate the adverse effects of ARNI.

## Conclusions

In conclusion, this review provides a thorough examination of the effectiveness of ARNI and its impact on renal outcomes in individuals with HF, including only RCTs. Sacubitril/valsartan has shown significant improvements compared to ACEis/ARBs in terms of decreased levels of NT-proBNP, avoidance of future decline in renal function, and reduced hospitalizations for HF. Notably, sacubitril/valsartan does not increase the risk of death from cardiovascular causes. Given the limited research available, we need larger-scale clinical trials to truly understand the long-term impact of angiotensin-neprilysin inhibition on renal function in HF patients.
